# Ultrafast 3D Bloch–Siegert B${}_{1}^{+}$‐mapping using variational modeling

**DOI:** 10.1002/mrm.27434

**Published:** 2018-10-12

**Authors:** Andreas Lesch, Matthias Schlöegl, Martin Holler, Kristian Bredies, Rudolf Stollberger

**Affiliations:** ^1^ Institute of Medical Engineering Graz University of Technology Graz Austria; ^2^ BioTechMed‐Graz Graz Austria; ^3^ Institute for Mathematics and Scientific Computing, Member of NAWI Graz University of Graz Graz Austria

**Keywords:** B${}_{1}^{+}$‐mapping, B${}_{1}^{+}$‐Bloch–Siegert shift, fast imaging, single breath hold acquisition, total generalized variation

## Abstract

**Purpose:**

Highly accelerated B${}_{1}^{+}$‐mapping based on the Bloch–Siegert shift to allow 3D acquisitions even within a brief period of a single breath‐hold.

**Theory and Methods:**

The B${}_{1}^{+}$ dependent Bloch–Siegert phase shift is measured within a highly subsampled 3D‐volume and reconstructed using a two‐step variational approach, exploiting the different spatial distribution of morphology and B${}_{1}^{+}$‐field. By appropriate variable substitution the basic non‐convex optimization problem is transformed in a sequential solution of two convex optimization problems with a total generalized variation (TGV) regularization for the morphology part and a smoothness constraint for the B${}_{1}^{+}$‐field. The method is evaluated on 3D in vivo data with retro‐ and prospective subsampling. The reconstructed B${}_{1}^{+}$‐maps are compared to a zero‐padded low resolution reconstruction and a fully sampled reference.

**Results:**

The reconstructed B${}_{1}^{+}$‐field maps are in high accordance to the reference for all measurements with a mean error below 1% and a maximum of about 4% for acceleration factors up to 100. The minimal error for different sampling patterns was achieved by sampling a dense region in *k*‐space center with acquisition times of around 10–12 s for 3D‐acquistions.

**Conclusions:**

The proposed variational approach enables highly accelerated 3D acquisitions of Bloch–Siegert data and thus full liver coverage in a single breath hold.

## 1 INTRODUCTION

At high and ultra high field (*B*_0_ ≥ 3 T) the magnitude of the transmit radio frequency (RF) field $|B_{1+}|$ can deviate substantially from the desired value. Therefore, an accurate measurement of $|B_{1+}|$ is essential for many applications in MRI, such as the design and calibration of multi‐transmit RF‐pulses^1^, ^2^ including B${}_{1}^{+}$‐shimming.^3^ In quantitative MRI it is crucial to correct for spatial flip‐angle variations influencing the idealized signal models, for example,^4^, ^5^ For these purposes many techniques were developed to determine accurate B${}_{1}^{+}$‐field maps. Due to the lack of diagnostic information fast acquisition is required.

One of the first techniques to map the B${}_{1}^{+}$‐field was the Double‐Angle‐Method (DAM),^6^, ^7^ which utilizes the magnitude ratio between two acquisitions with flip‐angles of *α* and 2*α*, respectively. However, this approach suffers from long acquisition times due to its inherent T_1_ dependency requiring long TR. To overcome this limitation improved versions of DAM were presented in^8^, ^9^ by “applying a B${}_{1}^{+}$‐insensitive magnetization reset at the end of each data acquisition”.^9^ Other magnitude based methods were presented like^10^ (AFI), exploiting the ratio of the steady‐state signal magnitude of two interleaved GRE‐acquisitions with different TR,^11^ which is based on the signal null at a certain flip‐angle or (DREAM by Nehrke et al.^12^) based on the ratio between a stimulated and an FID‐echo. In contrast to these works established on magnitude information other approaches exploiting the signal phase were proposed. One approach was presented by Morrell et al.^13^ using the phase information after two consecutive RF‐pulses along different axes. An improved version which is more robust against B_0_‐inhomogeneities was presented in.^14^ Nevertheless, both methods require very short RF‐pulses and are therefore restricted to non‐selective excitation.

A very promising phase‐based approach was presented by Sacolick et al.^15^ that exploits the shift in resonance frequency caused by an off‐resonant RF‐field—the so called Bloch–Siegert (BS) shift. This method was the first being able to directly measure the B${}_{1}^{+}$‐field and not only the flip‐angle *α*. Because this method is independent of T_1_ it does not suffer from long TR periods, however, an off‐resonant BS‐pulse with high magnitude and long duration is required to achieve a suitable signal. This increases the specific absorption rate (SAR) drastically and therefore limits the minimum possible TR due to patient safety constraints. As a result acquisition times in the order of minutes are necessary for volumetric measurements.

Two possibilities exist to further accelerate data acquisition. First, imaging strategies were proposed to acquire more data after one BS‐encoding pulse using faster read‐out strategies such as turbo spin echo (TSE),^16^ EPI‐readout^17^, ^18^ or spiral trajectories.^18^, ^19^ These approaches have, however, its own challenges at high and ultra‐high field strength. The second acceleration strategy is to acquire less Cartesian encodings, usually termed subsampling, and recover the missing information within the reconstruction using concepts of parallel imaging (PI) and compressed sensing (CS). Such an approach was proposed by Sharma et al.^20^ using a modified SPIRiT‐reconstruction,^21^ that yields acceleration factors of about 30 without sacrificing accuracy. This method already performs very well, but further improvement can be expected by directly applying a smoothness constraint to the reconstructed B${}_{1}^{+}$‐field. In Zhao et al.^22^ a Tikhonov regularization was applied to improve the B${}_{1}^{+}$‐field estimation especially in low signal regions in a multi‐transmit setting out of fully sampled BS‐data. In the case of subsampling this has not been done so far. Specific sampling patterns typically play an important role for the reconstruction of morphological images from subsampled data.^23^ Therefore we also investigated this influence for B${}_{1}^{+}$‐mapping in this study.

For single breath hold 3D‐B${}_{1}^{+}$‐mapping we propose a highly accelerated method based on the efficient reconstruction of subsampled BS‐data using a two‐step regularization strategy. This tailored regularization reflects the prior knowledge of piece‐wise smoothness on the underlying morphological image and the prior knowledge of spatial smoothness on the B${}_{1}^{+}$‐field. This allows us to exploit shared information present in the measured data. Evaluation of the resulting algorithm is carried out with retrospectively and prospectively accelerated in vivo measurements against fully sampled BS‐data. Furthermore, we demonstrate abdominal in vivo acquisition with full liver coverage during a single breath hold.

## 2 THEORY

### 2.1 Bloch Siegert approach for B${}_{1}^{+}$‐mapping

By applying an RF‐field with arbitrary resonance‐offset *ω*_BS_, a slight shift in resonance frequency can be observed in any NMR experiment.^24^ This effect depends on the RF‐magnitude and was exploited in^15^ to map the spatial varying B${}_{1}^{+}$‐field by applying an off‐resonant RF‐pulse (BS‐pulse) between excitation and readout in an arbitrary MRI sequence, causing a B${}_{1}^{+}$‐ dependent phase shift *ϕ*_BS_ after that pulse in each voxel. Under the assumption *ω*_BS_ ≫ *γB*_1_ 15 this additional phase shift only depends on the spatially varying squared B_1_ peak‐magnitude $B_{1,\text{peak}}^{2}$ and a pulse‐shape dependent constant *K*_BS_. The factor *K*_BS_ can be computed as a function of the normalized BS‐pulse‐shape *B*_1,norm_(*t*) with duration $T_{\text{pulse}}$, the resonance‐offset *ω*_BS_ and the gyro‐magnetic ratio *γ* leading to the following equation for the phase shift *ϕ*_BS_ (see Equation (6) in^15^):

(1) $$\phi_{\text{BS}}\;=\;B_{1,\text{peak}}^{2}\int_{0}^{T_{\text{pulse}}}\frac{{\left(\gamma B_{1,\text{norm}}\left(t\right)\right)}^{2}}{2\omega_{\text{BS}}\left(t\right)}dt\;=\;B_{1,\text{peak}}^{2}\cdot K_{\text{BS}}$$

To separate the phase shift *ϕ*_BS_ from other effects influencing the signal phase (background phase) such as B_0_‐field inhomogeneities, receiver coils or excitation, a reference measurement is required, which is typically performed as an acquisition with the negative resonance‐offset −*ω*_BS_ to increase the signal‐to‐noise ratio (SNR) in the final B${}_{1}^{+}$‐ map. The signals of these two acquisitions, *I*_+_ for positive and *I*_−_ for the negative resonance‐offset, are proportional to the magnitude of the magnetization *M*, the background phase *ϕ*_0_ and the desired BS‐phase *ϕ*_BS_.

(2) $$I_{+}\propto \left|M\right|e^{j\left(\phi_{0}+\phi_{\text{BS}}\right)}$$

(3) $$I_{-}\propto \left|M\right|e^{j(\phi_{0}-\phi_{\text{BS}})}$$

The B${}_{1}^{+}$‐map is given as the peak value of the applied BS‐pulse *B*_1,peak_. It can be calculated easily out of these two measurements in the fully sampled case by a complex division of the independently reconstructed images, *I*_+_ and *I*_−_, by reformulation of Equation (1) under the assumption of temporally constant *ϕ*_0_ as follows:

(4) $$B_{1,\text{peak}}\;=\;\sqrt{\frac{\text{arg}\left(I_{+}/I_{-}\right)}{2K_{\text{BS}}}}\;=\;\sqrt{\frac{\phi_{\text{BS}}}{K_{\text{BS}}}}$$

### 2.2 Variational BS‐reconstruction from highly subsampled data

The proposed approach to accelerate the $B_{1}^{+}$‐mapping is to employ a tailored subsampling of Fourier data combined with variational image reconstruction. The two measurements with positive and negative resonance‐offset as described above yield subsampled Fourier data *k*_*j*+_ and *k*_*j*−_ corresponding to the two images $I_{+}\;\;≃\;\;\left|M\right|e^{j(\phi_{0}\;+\;\phi_{\text{BS}})}$ and $I_{-}\;\;≃\;\;\left|M\right|e^{j(\phi_{0}-\phi_{\text{BS}})}$, respectively. Our goal is to obtain the phase shift *ϕ*_BS_ from these measurements. Due to the subsampling, however, it is not possible to separate this phase shift from the magnetization |*M*| and the background phase *ϕ*_0_ directly in the measured *k*‐space data. To overcome this, we use a variational approach to recover the three unkonwn quantities from the subsampled Fourier data. To this aim, a direct approach would be to seek for morphological data $p\;≃\;|M|e^{j\phi_{0}}$ and phase shift data $q\;≃\;e^{j\phi_{\text{BS}}}$ with |*q*| = 1 given as solutions of the minimization problem

(5) $$\min_{\begin{array}{c} p,q\\ |q|\;=\;1 \end{array}}\frac{\lambda }{2}\sum_{j}^{N_{c}}\|P_{+}F\left(c_{j}pq\right)-k_{j+}{\|}_{2}^{2}\;+\;\frac{\mu }{2}\sum_{j}^{N_{c}}{\left\|P_{-}F\left(c_{j}p\bar{q}\right)-k_{j-}\right\|}_{2}^{2}\;+\;R_{1}\left(p\right)\;+\;R_{2}\left(q\right).$$

Here the first two terms match the pointwise products $pq\;≃\;|M|e^{j(\phi_{0}\;+\;\phi_{\text{BS}})}$ and $p\bar{q}\;≃\;\left|M\right|e^{j(\phi_{0}-\phi_{\text{BS}})}$, with *q* being the complex conjugate of *q*, to the corresponding acquired data *k*_*j*+_ and *k*_*j*−_, respectively. The last two terms employ a regularization of the morphological data *p* and the phase shift data *q*. The regularization parameters *λ* and *μ* control the influence of the data fidelity terms on the whole cost function in comparison to the regularization terms weighted identically with one. Their choice depends on the noise‐level and image resolution. The data fidelity terms are defined as the sum over the squared *L* ^2^‐norm in each of $N_{c}$ receiver channels *j*. The MR forward model is given by a point wise multiplication with the precalculated receiver coil‐sensitivity maps *c*_*j*_, the discrete Fourier operator F and the subsampling patterns *P*_+_ and *P*_−_ for each acquisition.

Although Equation (5) is a rather natural approach for the problem under consideration, it comprises the solution of a non‐convex optimization problem, also when using convex regularization terms *R*_1_ and *R*_2_. In particular, even if we would drop the non‐convex constraint |*q*| = 1, due to the mappings (*p*,*q*) ↦ *pq* and (*p*,*q*) ↦ *p* *q*, the data fidelity terms are still non‐convex. As a result, one can generally not expect to obtain a globally optimal solution of Equation (5).

To overcome the non‐convexity, we reformulate Equation (5) using a change of variables, where we define *u* = *pq* and $v\;=\;{\bar{q}}^{2}$. With these new variables, the data fidelity term in Equation (5) reads as

(6) $$\left(u,v\right)\;\mapsto \;\frac{\lambda }{2}\sum_{j}^{N_{c}}\|P_{+}F\left(c_{j}u\right)-k_{j+}{\|}_{2}^{2}\;+\;\frac{\mu }{2}\sum_{j}^{N_{c}}{\left\|P_{-}F\left(c_{j}uv\right)-k_{j-}\right\|}_{2}^{2}.$$

We see that the variable *v* only appears in the second term. Now adding two regularization terms for *u* and *v* instead of the ones on *p* and *q* as in Equation (5) would still yield a non‐convex problem which in particular comprises two data fidelities for *u*. However, if we drop the second data fidelity for *u* (which corresponds to using less measurements), the minimization problem for *u* decouples from the terms involving the variable *v*. This allows to separately first solve a convex variational problem for $u\;=\;pq\;≃\;|M|e^{j(\phi_{0}\;+\;\phi_{\text{BS}})}$ and afterward, having *u* fixed, a second convex variational problem for $v\;=\;{\bar{q}}^{2}\;≃\;e^{-j2\phi_{\text{BS}}}$, where we drop the non‐convex constraint |*q*| = 1 to obtain convexity. The phase shift *ϕ*_BS_ can be obtained directly from the optimizer $\hat{v}$.

The first step is realized by solving the convex minimization problem

(7) $$\hat{u}\;=\;\underset{u}{\arg\min}\frac{\lambda }{2}\sum_{j}^{N_{c}}{\left\|P_{+}F\left(c_{j}u\right)-k_{j+}\right\|}_{2}^{2}\;+\;{\text{TGV}}_{\alpha }^{2}\left(u\right),$$

where we employ the second order total generalized variation (TGV^2^) functional^25^ for regularization of the unknown *u*, which contains morphological information that is modulated by a smooth phase shift. The functional ${\text{TGV}}_{\alpha }^{2}$ is known to be a suitable image prior for morphological MR images since it enforces piece‐wise smooth solutions, which is exactly the behavior of MR images with edges at tissue boundaries and modulated excitation and receiver inhomogeneities. Its applicability in MRI was already demonstrated in^26^ for reconstruction from subsampled measurements, diffusion‐tensor imaging,^27^ quantitative‐susceptibility mapping^28^ or in joint MR‐PET reconstruction.^29^ The ${\text{TGV}}_{\alpha }^{2}$ functional is defined according to,^30^, ^31^ where the parameter *α* = (*α*_0_, *α*_1_) balances between first order derivative information and second order derivative information. The ratio $\frac{\alpha_{0}}{\alpha_{1}}$ is fixed to a value of $\sqrt{2}$ in the 2D and to $\sqrt{3}$ in the 3D case, which was found to be a robust choice^32^ in image reconstruction.

The second step of obtaining the phase information is realized via the solution of the convex optimization problem

(8) $$\hat{v}\;=\;\underset{v}{\arg\min}\frac{\mu }{2}\sum_{j}^{N_{c}}\|P_{-}F\left(c_{j}\hat{u}v\right)-k_{j-}{\|}_{2}^{2}\;+\;\frac{1}{2}{\left\|\nabla v\right\|}_{2}^{2}.$$

Here, the unknown *v* corresponds to the B${}_{1}^{+}$‐field, which is known to be spatially smooth, and hence the squared *L* ^2^‐norm of the image gradient is used for regularization (H1‐regularization).^33^

Overall, this yields to a two‐step reconstruction method that comprises the sequential solution of two convex optimization problems such that the optimizer $\hat{v}\;\approx \;e^{-j2\phi_{\text{BS}}}$ of the second optimization problem exhibits a phase equal to the doubled BS‐phase *ϕ*_BS_ without morphological structure leading to

(9) $$B_{1,\text{peak}}\;=\;\sqrt{\frac{-\arg(\hat{v})}{2K_{\text{BS}}}}.$$

## 3 METHODS

### 3.1 Implementation

#### 3.1.1 Numerical solution

The optimization problem in Equation (7) of the proposed two‐step algorithm belongs to the class of non‐smooth convex optimization problems that can be solved efficiently with the primal‐dual splitting algorithm proposed in.^34^ The specific adaption of Equation (7) to the primal‐dual framework is described in.^26^ The optimization problem in the second step (Equation (8)) is a smooth and convex problem that can be solved using the well‐known conjugate‐gradient (CG) algorithm^35^ on the normal equations. Defining the linear forward operator in Equation (8) as $K:v\;\mapsto \;{\left(P_{-}F\left[c_{j}\hat{u}\cdot v\right]\right)}_{j\;=\;1,\;\cdots ,\;N_{c}}$, this accounts to solve

(10) $$\mu K^{H}\left(Kv-k_{-}\right)\;+\;\nabla^{H}\nabla v\;=\;0,$$

or equivalently

$$\left(\mu K^{H}K-\Delta \right)v\;=\;\mu K^{H}k_{-}.$$

#### 3.1.2 Reconstruction framework

The overall reconstruction framework was implemented in MATLAB (MathWorks, Inc., Natick, Massachusetts). To reduce the calculation time, the iterative optimization for Equations (7) and (8) were implemented in C++/CUDA (NVIDIA Corporation, Santa Clara, CA) using a modified version of an open‐source GPU‐library (AGILE)^36^ and a reconstruction library (AVIONIC).^37^ Receiver coil‐sensitivities were estimated from the fully sampled *k*‐space data using the method proposed by Walsh et al.,^38^ that was also used for coil combination to calculate the fully sampled reference and the zero padded low resolution estimates from the multi‐coil measurements. We further note that data normalization was carried out with respect to the maximum of a Hamming‐filtered low resolution estimate from the positive BS‐dataset similar to.^39^ The reconstruction framework with examples can be found online at “https://github.com/IMTtugraz/BSReconFramework”.

### 3.2 Validation and parameter optimization

To assess the practical applicability of the developed algorithm, different in vivo investigations from healthy volunteers with retrospective and prospective subsampling have been performed.

#### 3.2.1 In vivo measurements

All in vivo measurements were gained from five male healthy volunteers in the age between 28 and 33 with the approval of the responsible ethics committee on a Skyra 3 T system (Siemens, Erlangen, Germany). To measure the BS‐shift a GRE sequence was modified by adding an off‐resonant Gaussian shaped RF‐pulse between excitation and readout as stated in^15^ with a duration $T_{\text{pulse}}\;=\;10$ ms, an off‐resonance frequency *f*_BS_ = 4 kHz and an on‐resonant equivalent flip‐angle *α*_BS_ = 1000^∘^ leading to a pulse constant *K*_BS_ = 53.4rad/G^2^. Measurement data was acquired using a 20‐channel head/neck receive‐coil (Siemens, Erlangen, Germany) and the birdcage body‐coil for transmit. The acquired in vivo 3D brain datasets have a matrix size of 128 × 128 × 32 as in,^20^ a squared FOV with 230 mm, a resolution of 2 mm in slice direction and a slice oversampling of 25%. TE and TR were set to minimal values of TE/TR = 13.5/95 ms, respectively, and an excitation flip‐angle of *α* = 25^∘^ was used which corresponds to the mean Ernst‐angle in gray and white matter. The minimal TE is restricted by the length of the BS‐pulse and the TR by the SAR‐constraint. The acquisition parameters for liver and knee dataset were adjusted to a matrix size of 128 × 128 × 44 and 128 × 128 × 52, a FOV of 220 and 150 mm and a resolution in slice direction of 3.2 and 2.5 mm, respectively.

#### 3.2.2 Error evaluation

The reconstructed B${}_{1}^{+}$‐maps *B*_1rec_ were validated against a reference map *B*_1ref_, which is a fully sampled dataset. The error maps are defined as $|B_{1\text{ref}}-B_{1\text{rec}}|/B_{1\text{nom}}$ with a normalization to the desired B_1_ magnitude *B*_1nom_ necessary to achieve the nominal flip‐angle *α*_BS_. This error measure is proportional to the correction error in many quantitative MRI models. Each result is further evaluated as mean absolute error (MAE), its median value (medAE) and the 99% quantile *q*_99%_ over a certain region of interest (ROI) covering the whole brain inside the cranial bone structure. For the evaluation of random subsampling patterns all three error measures are given as average over 10 independent trials. The reconstruction results were compared to the fully sampled reference and a low resolution estimate, which is obtained as zero padded inverse FFT with subsequent coil combination for both measurements using^38^ and Equation (4). Furthermore, results are evaluated by an error histogram.

#### 3.2.3 Tuning of the regularization parameters

In our proposed approach we need to tune two regularization parameters *μ* and *λ* in order to achieve optimal results. For that purpose we first performed a grid search for one particular measured dataset and subsampling pattern. Those led to the minimum MAE were fixed for all further experiments. The found values are *λ*_*opt*_ = 64 and *μ*_*opt*_ = 5.0 · 10^−4^.

#### 3.2.4 Subsampling patterns

The in vivo data were retrospectively subsampled from the fully sampled reference *k*‐space data. Initially, we used a rectangular region in *k*‐space center (block pattern) as subsampling pattern which is defined by a fixed number of *n*×*m* Cartesian encodings in *k*_*y*_ and *k*_*z*_ phase encoding direction. Results with this type of pattern were already shown in.^40^ The block sampling strategy was used, because the B${}_{1}^{+}$‐dependent information is mostly encoded with low spatial frequency information. However, in compressed sensing image reconstruction, it is common to use irregular subsampling patterns, to fulfill the incoherence condition. Therefore, randomized sampling schemes as proposed in the seminal work of Lustig et al.^41^ were investigated. Therein, random samples are generated according to polynomial density kernels around the *k*‐space center. In order to generate more densely sampled patterns we also substituted the polynomial kernels with Gaussian density kernels. Block and Gaussian patters are schematically visualized in Supplementary Figure S1. The benefit of random sampling was explored firstly, for only the positive and secondly for both, positive and negative, BS‐pulse encoding as described in.^42^

The random pattern described in^41^ is defined by the parameter *p*, which is the polynomial degree used in the density function. A higher *p*‐value means that the sampling points are spread more uniformly over the whole *k*‐space. The Gaussian density pattern is described by its standard deviation *σ*_*y*_ and *σ*_*z*_ in both phase encoding directions. The effect of such patterns with different distribution parameters and a fixed acceleration factor *R* were evaluated against the fully sampled reference. For the acquisition of prospectively subsampled data the sequence was modified, such that only an adjustable number of Cartesian encodings are acquired in *k*‐space center in both phase encoding directions.

## 4 RESULTS

**Figure 1:**
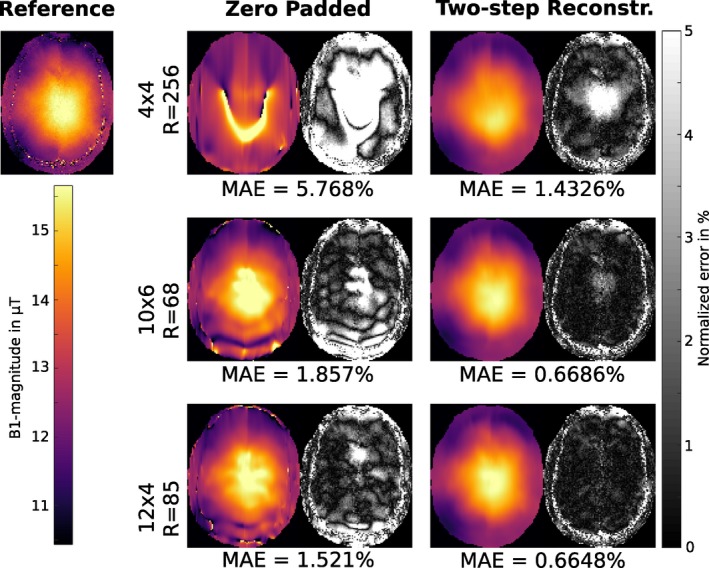
*Retrospectively subsampled:* B${}_{1}^{+}$‐map in *μ*T for fully sampled reference, low resolution estimate and the result of the proposed two‐step reconstruction method for a retrospectively subsampled dataset in the brain of a healthy volunteer for a block size of 4 × 4, 10 × 6 and 12 × 4 encodings in the *k*‐space center. The right part of each column shows the error map for the corresponding result as normalized error in percent of the desired B_1_ peak‐magnitude. The MAE is given as the mean of the error map over the whole 3D‐brain inside the cranial bone structure for each case

Figure 1 shows results of the proposed two‐step reconstruction method on retrospectively subsampled measurement data in the brain for different block sizes. The results are compared to the fully sampled reference and a low resolution estimate with the same amount of data. The B${}_{1}^{+}$‐maps gained by zero padding (low resolution) are highly corrupted with artifacts especially in low signal regions where dominant phase jumps are likely to occur. In contrast to that the two‐step reconstruction method yields artifact free results in very good accordance to the fully sampled reference for block sizes with 10 × 6 and 12 × 4 encodings and even for a block size of 4×4 the error is bounded to comparable low values. In Table 1 MAE, medAE and *q*_99%_ values for different block sizes are summarized for both methods. All three error measures show a substantial improvement for our two‐step reconstruction approach compared to results gained from low resolution data for all cases.

**Table 1:** *Retrospectively subsampled:* MAE, medAE and *q*_99%_ inside the described ROI for different block sizes in percent of the desired B_1_ peak‐magnitude and the corresponding acceleration factor *R*. The values are given for the low resolution estimate and the result of the proposed two‐step reconstruction method

			Zero Padded		Two‐step Reconstruction		
pattern	R	MAE (%)	medAE (%)	*q*_99%_ (%)	MAE (%)	medAE (%)	*q*_99%_ (%)
4 × 4	256.0	5.768	3.124	46.726	1.433	1.062	6.518
6 × 4	170.7	3.834	2.529	21.843	0.938	0.700	4.220
6 × 6	113.8	3.534	2.452	18.810	0.846	0.637	3.748
8 × 4	128.0	2.956	1.973	17.881	0.812	0.622	3.350
8 × 6	85.3	2.699	1.824	16.811	0.747	0.564	3.216
8 × 8	64.0	2.681	1.799	17.651	0.728	0.549	3.121
10 × 4	102.4	2.049	1.482	9.154	0.731	0.558	3.084
10 × 6	68.3	1.857	1.366	8.187	0.669	0.505	2.891
12 × 4	85.3	1.521	1.139	6.779	0.665	0.512	2.799
12 × 6	56.9	1.416	1.039	6.511	0.609	0.463	2.593
12 × 12	28.4	1.321	0.968	5.672	0.573	0.437	2.417

**Figure 2:**
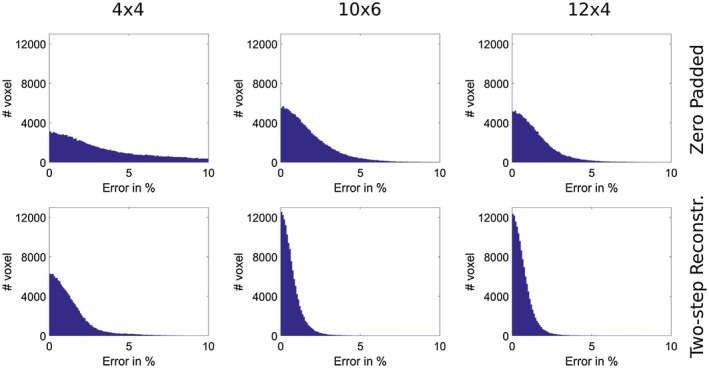
*Retrospectively subsampled:* Error histogram for the retrospectively subsampled dataset compared to the fully sampled reference in percent of the desired B_1_ peak‐magnitude for block sizes of 4 × 4, 10 × 6 and 12 × 4 encodings in the *k*‐space center. The error histograms are shown for zero padded low resolution estimate and the result of our proposed two‐step reconstruction method

Figure 2 displays error histograms for the low resolution estimate and the proposed two‐step reconstruction method to visualize the error distribution inside the described ROI. The histograms are shown for a retrospectively subsampled dataset with block sizes of 4 × 4, 10 × 6 and 12 × 4 encodings in the *k*‐space center. The error histograms for the proposed two‐step reconstruction are much narrower for all block sizes as compared to zero padded results. Using the proposed two‐step reconstruction method about 1.4%/1.7%/11% of all voxels exceed a defined error limit of 2.5% for pattern sizes of 12 × 4, 10 × 6 and 4 × 4 compared to 16%/23%/55% using zero padding.

**Figure 3:**
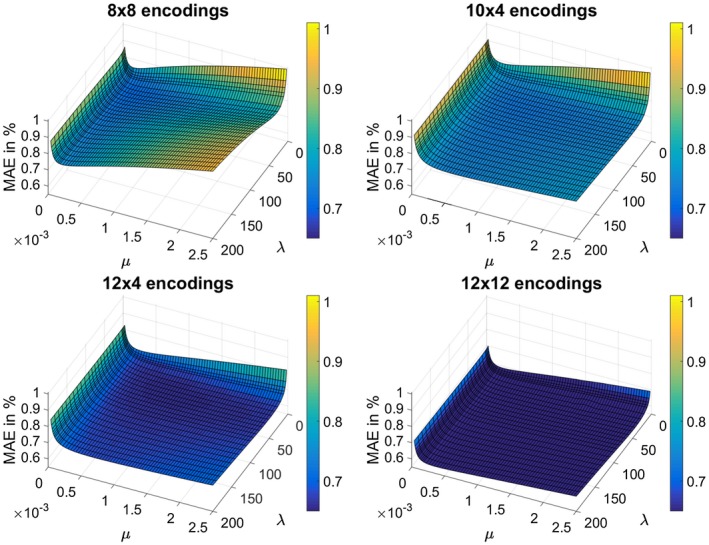
MAE inside the described ROI as a function of both regularization parameters *λ* and *μ* for different block sizes (8 × 8, 10 × 4, 12 × 4 and 12 × 12 encodings in *k*‐space center) in percent of the desired B_1_ peak‐magnitude. For this evaluation the retrospective subsampled brain dataset shown in Figure 1 was used

Figure 3 shows the MAE value for different block sizes as a function of the regularization parameters *μ* and *λ*. The error stays stable over a wide range, reflecting the algorithm's robustness to non‐optimally tuned regularization parameters. Furthermore, for those sampling patterns where the block size has a similar ratio of both phase encoding directions as the imaging matrix (12 × 4 and 10 × 4 encodings in *k*‐space center) a lower sensibility with respect to changes in *λ* and *μ* can be observed.

**Figure 4:**
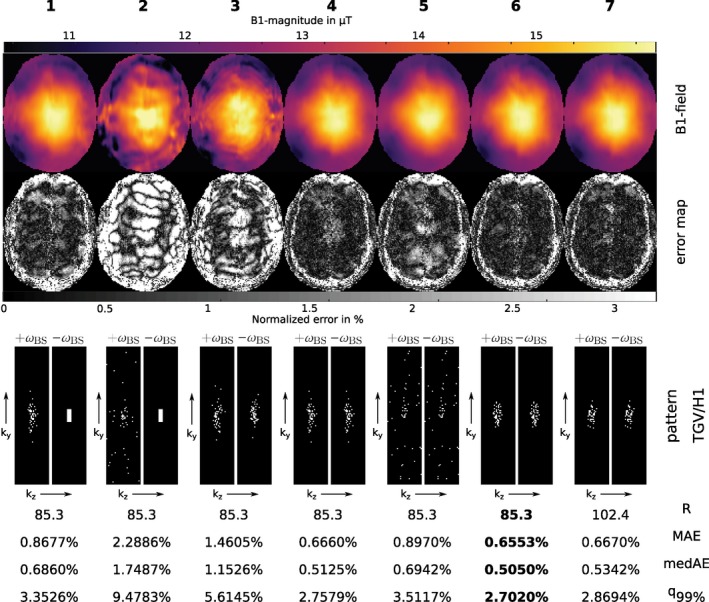
*Retrospectively subsampled:* First row: Reconstruction results of the proposed two‐step reconstruction method in *μ*T using different subsampling patterns. Second row: Corresponding error maps as normalized error in percent of the desired B_1_ peak‐magnitude (Reference see Figure 1). Third row: Combinations of subsampling patterns for the first step *P*_+_ (TGV, +*ω*_BS_, left pattern) and the second step *P*_−_ (H1, −*ω*_BS_, right pattern) were investigated as follows: Case 1 and 2: 12 × 4 block‐pattern in *k*‐space center in H1‐part and a variable density pattern out of^41^ in TGV‐part with *p* = 14.4 and *p* = 25 respectively. Case 3: Different instances of this pattern with *p* = 14.4 in both parts. Case 4 and 5: The same instances of this pattern with *p* = 14.4 and *p* = 25 respectively. Cases 6 and 7: Pattern with Gaussian density function with *σ*_*y*_ = 5 and *σ*_*z*_ = 2, in case 7 with a higher acceleration factor. For each case the achieved acceleration *R*, the MAE, the medAE and the *q*_99%_ quantile inside the described ROI are given in percent of the desired B_1_ peak‐magnitude as mean over 10 trails with different realizations out of the described probability distribution

Figure 4 shows results for the two‐step reconstruction method for different irregular subsampling patterns. Pattern combinations that irregularly sample higher frequency information in the TGV‐part *P*_+_ while only densely sampling the *k*‐space center in the H1‐part *P*_−_ (patterns 1 and 2) did not improve the reconstruction quality but lead to an increase in error, especially if the distribution favors sampling higher spatial frequencies. Using two different irregular sampling patterns with the same distribution parameters in both parts of the reconstruction also introduces artifacts. Increased reconstruction quality is achievable when the same irregular pattern is used in both reconstruction steps as it is done in cases 4–7, where the error decreases the more sampling is concentrated around the *k*‐space center. The highest concentration is achieved using a Gaussian density function (pattern 6) which yielded the highest B${}_{1}^{+}$‐accuracy. Furthermore, the Gaussian density function allows even higher acceleration with only slight increase in error (pattern 7). Compared to the best block‐sampling pattern (12 × 4 encodings in Figure 1) a slight improvement in error with equal acceleration rate *R* can be observed, nevertheless for the acquisition of prospectively subsampled data block‐sampling was used to keep the acquisition protocol simple.

**Figure 5:**
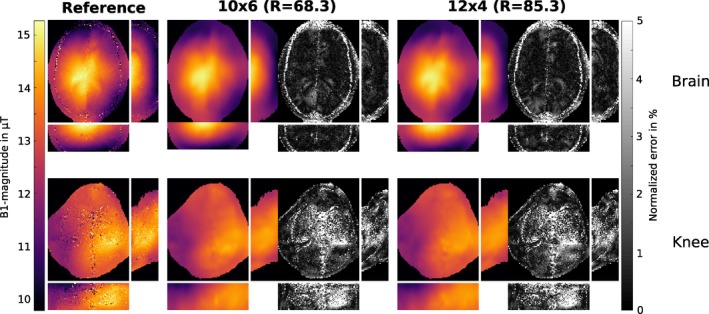
*Prospectively subsampled:* B${}_{1}^{+}$‐map in *μ*T for fully sampled reference and the result of our proposed two‐step reconstruction method with 10 × 6 and 12 × 4 encodings in the *k*‐space center and the corresponding error map in percent of the desired B_1_ peak‐magnitude from prospectively subsampled data. The B${}_{1}^{+}$‐maps are shown for a brain and knee dataset from two different healthy volunteers. All results are shown in a transverse, coronal and sagittal orientation

**Figure 6:**
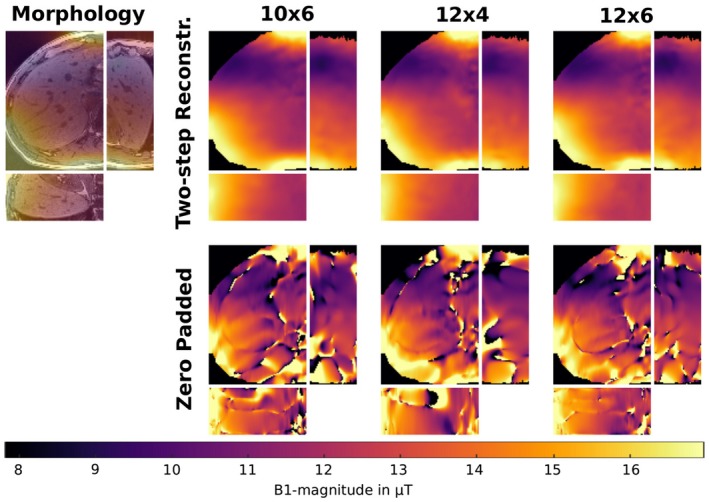
*Prospectively subsampled:* B${}_{1}^{+}$‐map in *μ*T as result of our proposed two‐step reconstruction method with 10 × 6, 12 × 4 and 12 × 6 encodings in the *k*‐space center in the liver of a healthy volunteer. These results are compared to those gained with zero padding using the same amount of data. The datasets were measured prospectively subsampled and acquired in a single breath hold. Due to the lack of a reference in the liver dataset, the reconstructed B${}_{1}^{+}$‐map (10 × 6 encodings) is also shown as an overlay to a morphological scan to show the underlying morphological structure. All results are shown in a transverse, coronal and sagittal orientation

Figures 5 and 6 show results obtained with the proposed two‐step reconstruction approach from prospectively subsampled brain, knee and liver datasets from different healthy volunteers. For the brain and knee dataset we further provide an additional fully sampled dataset as reference. For the liver dataset it is not feasible to obtain a fully sampled reference due to breath hold limitations such that an overlay of the B${}_{1}^{+}$‐field on a morphological scan is provided. To further show the improvement of the proposed method over zero padding Figure 6 also provides zero padded results of the liver dataset for comparison. Zero padded results for brain and knee dataset are further shown in Supplementary Figure S2. For brain and knee dataset the results of our two‐step reconstruction are in high agreement with the fully sampled reference, whereas the knee dataset shows a slight corruption due to high blood flow in the leg artery that leads to phase errors. The effect is much more prominent in the reference than in the final results of the proposed method. For the abdominal dataset in Figure 6 some minor heart‐motion related artifacts outside the liver tissue are visible.

## 5 DISCUSSION

In this work we presented a variational two‐step approach to reconstruct the B${}_{1}^{+}$‐field from highly subsampled BS‐data. Different subsampling strategies were investigated on the basis that the spatially smooth B${}_{1}^{+}$‐field information mostly relies on low spatial frequencies in the *k*‐space center. In the initial hypothesis we assumed that it might be advantageous to sample a broader distribution of spatial frequencies for the reconstruction of the TGV^2^ regularized part *I*_+_ to better characterize the morphological basis. The performed subsampling experiments, however, showed that it is more important to sample a dense *k*‐space center region for both BS‐acquisitions and to encode in both measurements the identical *k*‐space lines. This behavior can be probably explained that subsampling artifacts depend on the specific encoding pattern and their suppression is more effective for similar occurrence in both parts of the reconstruction. It could be shown that two different instances of a random pattern with the same distribution parameters exhibits substantially more artifacts in the final B${}_{1}^{+}$‐map compared to the reconstruction results using the identical encoding pattern. The investigation of the error for different distributed sampling patterns showed a flat minimum for a compact sampling in the *k*‐space center. The retrospective subsampling study (see Figure 1) suggest that a compact sampling with a block‐pattern of similar ratio as the imaging matrix yields nearly as good results as with Gaussian distributed dense sampling (see Figure 4) in terms of accuracy and achievable acceleration. Thus, the block sampling approach was implemented for in vivo measurements to simplify the acquisition protocol.

In contrast to this work, the authors in^20^ used the SPIRiT method^21^ to perform a joint reconstruction with staggered pattern, the acceleration potential was only investigated on top of a fixed number (20 × 20, 32 × 32) of auto calibration lines in a multi‐transmit system. Since the proposed algorithm also includes the principles of parallel imaging it relies on the precomputation of receiver coil‐sensitivities. For high acceleration factors this translates to an increased error when the highly subsampled BS‐data is used for estimation. It is then recommended to either use prescan calibration data or a concurrently measured dataset after the BS‐calibration scan. The application of the proposed method to multi‐transmit data and different *k*‐space trajectories is straight‐forward and will be subject to future research. A question which may arises is the ability to capture very localized B${}_{1}^{+}$‐field variations occurring for parallel transmit coils or at higher field strength. To give an idea of the behavior the experiment in Supplementary Figure S3 shows the B${}_{1}^{+}$‐map in a phantom placed very close to the elements of a small‐animal birdcage coil. Near these elements very localized B${}_{1}^{+}$‐field inhomogeneities occur which can be captured quite well with the proposed method. Nevertheless, depending on a specific coil configuration a detailed examination of the undersampling pattern would be necessary, which might lead to larger block sizes.

The investigation concerning the dependency of the reconstruction quality on the model parameters *λ* and *μ* showed that these are fairly stable over a wide range and across different pattern sizes for a given SNR scenario. Since the SNR is usually only altered slightly it is possible to achieve robust reconstruction results without additional tuning. GPU powered reconstruction on a NVIDIA Geforce Titan Xp GPU takes about 30 s for the complete 3D‐measurement.

General limitations of the BS‐method are phase drifts or phases fluctuations between positive and negative BS‐encoding. Although the used interleaved acquisition scheme^43^ makes the method more robust against phase drifts, phase fluctuations may still be an issue within regions with fast and pulsatile phase changes such as large arteries. In Figure 6 this becomes visible, for example, in the right part of the liver dataset due to heart motion or in Figure 5 within the knee dataset due to blood flow in the leg artery. Since the proposed method enforces smoothness on the B${}_{1}^{+}$‐field the error due to local disturbances is effectively suppressed and interpolated based on the local neighborhood in the resulting B${}_{1}^{+}$‐map (see knee dataset in Figure 5). In Figures 1 and 5 the described interpolation effect leads to an alleged increased error in the cranial bone structure where the fully sampled reference exhibits low signal leading to an uncertainty in the reference map. Therefore, this region was excluded from the error analysis.

In this work, we also performed an investigation about the feasible acceleration potential for 3D acquisitions and the expectable error in the B${}_{1}^{+}$‐field with respect to the fully sampled reference. For all investigated regions receiving array coils with 20–32 active coils were used. For these applications acceleration factors from 80 to 100 were achieved that reduces the acquisition time into the range of 10–12 s for the whole 3D‐dataset. From retrospective subsampling experiments mean errors below 1% and maximal errors below 4% were observed for the investigated setting and used acceleration factors. Further acceleration is achievable for a higher number of independent receiver coils or by sacrificing accuracy.

Similar acquisition times for whole brain coverage are still possible by a combination of BS‐based B${}_{1}^{+}$‐mapping with spiral readouts and optimized BS‐pulses (12 s)^18^, ^19^ or below 40 s combined with EPI readout.^17^ However, these methods are prone to artifacts in particular at high and ultrahigh field strength. Nevertheless, the combination of the proposed method with such trajectories is straight‐forward and further acceleration can be expected. For regions that allow a long readout train 3D‐single‐shot acquisition might be feasible. However, in this work we focused on the robust implementation of accelerated BS‐mapping for widely available Cartesian imaging.

In a recent work^44^ a method is described, where interleaved acquisition and ECG‐triggering are combined in a proper way to acquire cardiac B${}_{1}^{+}$‐maps. By a combination of this approach and the proposed method a 3D cardiac B${}_{1}^{+}$‐map in a few heart beats seems possible.

## 6 CONCLUSIONS

A new highly accelerated 3D B${}_{1}^{+}$‐mapping method based on the BS‐shift and reconstruction by variational modeling was introduced. The method is able to reconstruct 3D B${}_{1}^{+}$‐maps from parallel acquired Cartesian encodings within a typical breath‐hold period of a patient using acceleration factors of up to 100. With Cartesian encoding the method is stable even at very high field strength. The reconstruction errors were estimated from retrospective subsampling experiments and were found to be below 1% in mean and 4% in maximum.

## Supporting information


**Figure S1** Schematic representation of the block pattern and the irregular pattern with Gaussian density function. For the block pattern a rectangular region in *k*‐space center with a predefined number of *n* × *m* Cartesian encodings in *k*_*y*_ and *k*_*z*_, respectively is used. The irregular pattern with Gaussian density function is defined by the standard deviation *σ*_*y*_ and *σ*_*z*_ in both phase encoding directions. Here the ±2*σ*_*y*,*z*_ area is shown in red. The sampling pattern is gained by selecting a random number of Cartesian encodings according to the probability density function. The readout direction is *k*_*x*_ in all cases
**Figure S2** *Prospectively subsampled:* B${}_{1}^{+}$‐map in *μ*T for fully sampled reference and the **zero padded results** with 10 × 6 and 12 × 4 encodings in the *k*‐space center and the corresponding error map in percent of the desired B_1_ peak‐magnitude from prospectively subsampled data. The B${}_{1}^{+}$‐maps are shown for a brain and knee dataset from two different healthy volunteers. All results are shown in a transverse, coronal and sagittal orientation
**Figure S3** *Prospectively subsampled:* B${}_{1}^{+}$‐map in *μ*T for fully sampled reference and the proposed two‐step reconstruction method measured with a block size of 12 × 4. The measurement was performed with a TX/RX small‐animal birdcage coil with an inner diameter of 4 cm. The cylindrical agar phantom was placed very close to the elements of the birdcage, leading to localized B${}_{1}^{+}$‐field variations similar as in a parallel transmit setting. The measurement was performed using a FOV of 40 mm and a flip angle *α* = 12^∘^. To achieve optimal reconstruction results for this special case the regularization parameters have to be retuned, leading to the following values: *λ* = 5, *μ* = 16 · 10^−4^
