# Supplementary material for: Ultrafast 3D Bloch–Siegert B1+‐mapping using variational modeling
Source: Magn Reson Med. 2018 Oct 12;81(2):881–92. doi: 10.1002/mrm.27434 (PMC6491998; doi:10.1002/mrm.27434)
Supplement: Supplementary file 1 — Figure S1 Schematic representation of the block pattern and the irregular pattern with Gaussian density function. For the block pattern a rectangular region in k‐space center with a predefined number of n × m Cartesian encodings in k y and k z, respectively is used. The irregular pattern with Gaussian density function is defined by the standard deviation σ y and σ z in both phase encoding directions. Here the ±2σ y,z area is shown in red. The sampling pattern is gained by selecting a random number of Cartesian encodings according to the probability density function. The readout direction is k x in all cases Figure S2 Prospectively subsampled: B1+‐map in μT for fully sampled reference and the zero padded results with 10 × 6 and 12 × 4 encodings in the k‐space center and the corresponding error map in percent of the desired B1 peak‐magnitude from prospectively subsampled data. The B1+‐maps are shown for a brain and knee dataset from two different healthy volunteers. All results are shown in a transverse, coronal and sagittal orientation Figure S3 Prospectively subsampled: B1+‐map in μT for fully sampled reference and the proposed two‐step reconstruction method measured with a block size of 12 × 4. The measurement was performed with a TX/RX small‐animal birdcage coil with an inner diameter of 4 cm. The cylindrical agar phantom was placed very close to the elements of the birdcage, leading to localized B1+‐field variations similar as in a parallel transmit setting. The measurement was performed using a FOV of 40 mm and a flip angle α = 12∘. To achieve optimal reconstruction results for this special case the regularization parameters have to be retuned, leading to the following values: λ = 5, μ = 16 · 10−4 [file MRM-81-881-s001.pdf]

SUPPORTING INFORMATION FOR ONLINE PUBLICATION

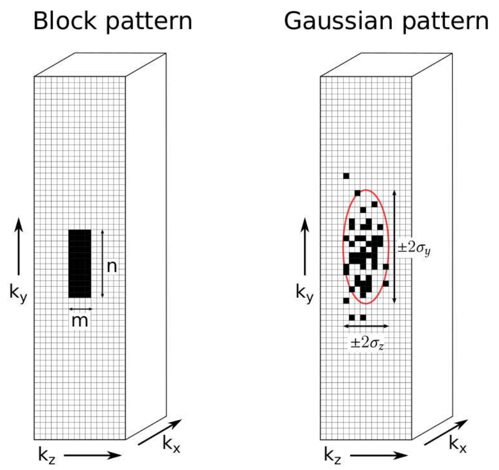

Supplementary Figure S1: Schematic representation of the block pattern and the irregular pattern with Gaussian density function. For the block pattern a rectangular region in k-space center with a predefined number of $n \times m$ Cartesian encodings in k_y and k_z, respectively is used. The irregular pattern with Gaussian density function is defined by the standard deviation σ_y and σ_z in both phase encoding directions. Here the $\pm 2\sigma_{y,z}$ area is shown in red. The sampling pattern is gained by selecting a random number of Cartesian encodings according to the probability density function. The readout direction is k_x in all cases.

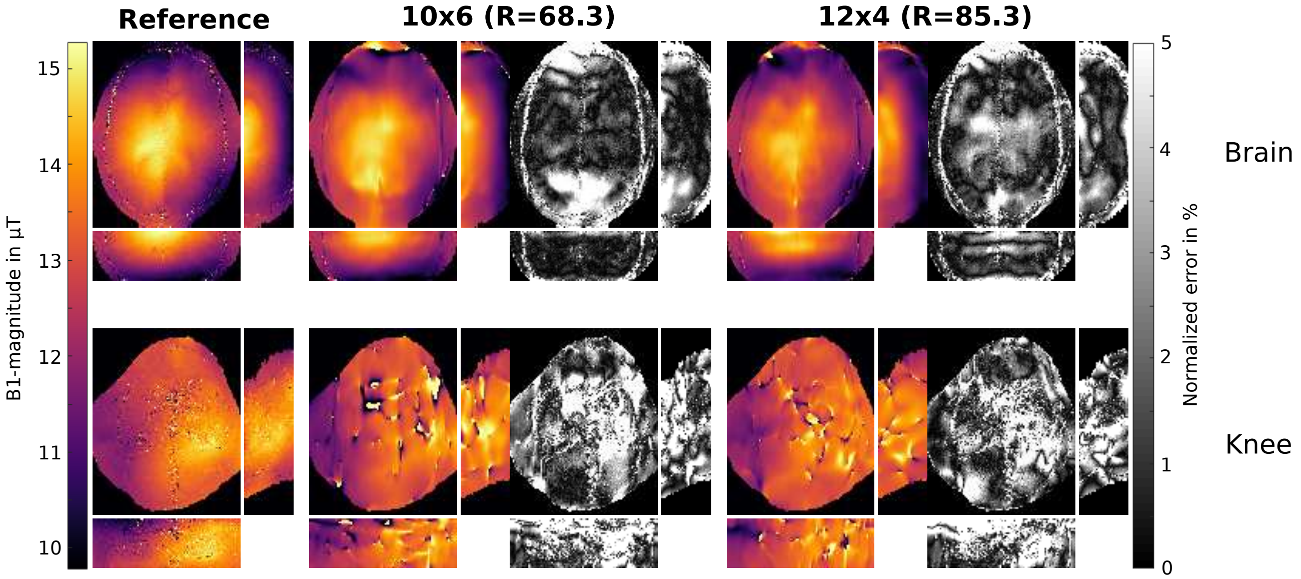

Supplementary Figure S2: *Prospectively subsampled:* $B_1{}^+$-map in µT for fully sampled reference and the **zero padded results** with 10×6 and 12×4 encodings in the k-space center and the corresponding error map in percent of the desired B_1 peak-magnitude from prospectively subsampled data. The $B_1{}^+$-maps are shown for a brain and knee dataset from two different healthy volunteers. All results are shown in a transverse, coronal and sagittal orientation.

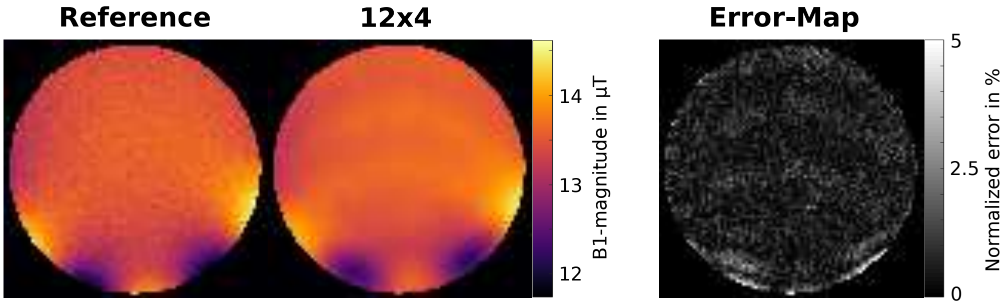

Supplementary Figure S3: *Prospectively subsampled:* $B_1{}^+$-map in µT for fully sampled reference and the proposed two-step reconstruction method measured with a block size of 12×4. The measurement was performed with a TX/RX small-animal birdcage coil with an inner diameter of 4 cm. The cylindrical agar phantom was placed very close to the elements of the birdcage, leading to localized $B_1{}^+$-field variations similar as in a parallel transmit setting. The measurement was performed using a FOV of 40 mm and a flip angle $\alpha = 12°$. To achieve optimal reconstruction results for this special case the regularization parameters have to be retuned, leading to the following values: $\lambda = 5$, $\mu = 16 \cdot 10^{-4}$
